# Design of a Thermoacoustic Sensor for Low Intensity Ultrasound Measurements Based on an Artificial Neural Network

**DOI:** 10.3390/s150614788

**Published:** 2015-06-23

**Authors:** Jida Xing, Jie Chen

**Affiliations:** 1 Faculty of Engineering, University of Alberta, Edmonton, AB T6G 2V4, Canada; E-Mail: jida1@ualberta.ca; 2 Canadian National Research Council National Institute for Nanotechnology, Edmonton, AB T6G 2M9, Canada

**Keywords:** Low Intensity Pulsed Ultrasound (LIPUS), thermoacoustic sensor, ultrasound intensity measurement, artificial neural network

## Abstract

In therapeutic ultrasound applications, accurate ultrasound output intensities are crucial because the physiological effects of therapeutic ultrasound are very sensitive to the intensity and duration of these applications. Although radiation force balance is a benchmark technique for measuring ultrasound intensity and power, it is costly, difficult to operate, and compromised by noise vibration. To overcome these limitations, the development of a low-cost, easy to operate, and vibration-resistant alternative device is necessary for rapid ultrasound intensity measurement. Therefore, we proposed and validated a novel two-layer thermoacoustic sensor using an artificial neural network technique to accurately measure low ultrasound intensities between 30 and 120 mW/cm^2^. The first layer of the sensor design is a cylindrical absorber made of plexiglass, followed by a second layer composed of polyurethane rubber with a high attenuation coefficient to absorb extra ultrasound energy. The sensor determined ultrasound intensities according to a temperature elevation induced by heat converted from incident acoustic energy. Compared with our previous one-layer sensor design, the new two-layer sensor enhanced the ultrasound absorption efficiency to provide more rapid and reliable measurements. Using a three-dimensional model in the K-wave toolbox, our simulation of the ultrasound propagation process demonstrated that the two-layer design is more efficient than the single layer design. We also integrated an artificial neural network algorithm to compensate for the large measurement offset. After obtaining multiple parameters of the sensor characteristics through calibration, the artificial neural network is built to correct temperature drifts and increase the reliability of our thermoacoustic measurements through iterative training about ten seconds. The performance of the artificial neural network method was validated through a series of experiments. Compared to our previous design, the new design reduced sensing time from 20 s to 12 s, and the sensor’s average error from 3.97 mW/cm^2^ to 1.31 mW/cm^2^ respectively.

## 1. Introduction

Low intensity pulsed ultrasound (LIPUS) has shown great utility and promise in medical therapeutic treatments, including bone and soft tissue healing, tooth root resorption [1,2,3,4], stem cell proliferation and differentiation [5,6], and antibody production [7,8,9]. In these applications, 1.5 MHz has been validated to be an effective stimulation frequency, and the applied intensities are within a range between 30 mW/cm^2^ and 100 mW/cm^2^. Accurate calibrations of ultrasound intensities have become important because treatment outcomes are highly dependent on the intensities and duration of LIPUS. If the ultrasound exposure level is too low, no biomedical or clinical effect will be observed, while too high a dose can cause adverse or damaging effects to the target tissues or cells [10,11]. Radiation force balance is a benchmark technique used to measure ultrasound power and intensity. However, a radiation force balance system is costly, difficult to operate, and compromised by measurement noise [12]. In an environment with noise vibration, the error of a radiation force balance for low intensity ultrasound measurement can easily surpass 20%. In a working environment such as in a biology laboratory, the measurement is likely to be affected by background vibrations produced from other lab equipment. In addition, the application of the radiation force balance is restricted in some situations. For example, an ultrasound holder with an array of transducers is commonly used in biological experiments to stimulate cells for various therapeutic applications. In this situation, it is difficult for the radiation force balance to directly measure the intensity of each individual transducer that has been fixed in the ultrasound holder, since it uses a large target to collect the ultrasonic beam which in turn would also collect the ultrasound beams of several other transducers simultaneously. Although the measurement may be realized by an absorber with a hole to isolate the each individual transducer by masking the other transducers, undoubtedly, it would also add to the complexity of the operation. Therefore, the development of a vibration-resistant, low-cost and easy-to-operate alternative measurement device is indispensable for rapid calibration of ultrasound intensity.

A vibration-resistant and cost-effective thermoacoustic sensor presents a suitable alternative to the radiation force balance. This type of sensor determines the ultrasound intensity according to the temperature rise caused by the heat produced from incident acoustic energy [13]. A sensor using the thermal method was developed to directly measure spatial-peak temporal-average intensity (I_spta_) or determine intensity beam profiles of ultrasonic fields [13,14,15], as it is commonly performed, for instance, in acoustic output characterization of diagnostic ultrasonic equipment for safety considerations. The thermoacoustic sensor is based on a conventional beam-plotting set-up in a water tank, which in principle can be used to calibrate a range of transducers. Different from spatial-peak temporal-average intensity (I_spta_), which represents the maximum intensity in the acoustic field, spatial-average temporal-average intensity (I_sata_) describes the average intensity of the acoustic field [16]. Therapeutic ultrasound applications require accurate measurements of spatial-average temporal-average (I_sata_) which cannot be directly measured by hydrophones or the above mentioned sensor. To this end, we designed a thermoacoustic sensor to measure I_sata_ of a commercial ultrasound generator, SonaCell (IntelligentNano Inc., Edmonton, AB, Canada), which generates low intensity pulsed ultrasound at a frequency of 1.5 MHz for therapeutic applications. We adopted a thermal method based on a transient temperature model in order to quickly measure ultrasound intensities [17,18]. To make the sensor easy to operate and maintain consistency during each measurement, we simplified the sensor set-up procedure using a concept of close-proximity [19] where the designed sensor is directly coupled to the transducer through the ultrasound medium (ultrasound gel or degassed water) to perform the measurements. In applying the close-proximity thermoacoustic sensor design for ultrasound calibration, an enhancement in response time, accuracy, and consistency of sensor measurements becomes critical. Therefore, further studies are still needed. This paper focuses on how to improve the performance of the sensor design through a new structural design and artificial neural network algorithm.

The performance of the thermal sensor heavily depends on the conversion efficiency from low intensity ultrasonic energy into heat. In our current study, we proposed a two-layer structure to increase the absorption efficiency of ultrasound energy. The first layer is a cylindrical plexiglass absorber, the same as our previous design [19]. A second layer is made of polyurethane rubber with a high attenuation coefficient to absorb extra ultrasound energy. This design provides higher conversion efficiency than our previous one-layer design. The detailed configuration is discussed in section two.

The thermoacoustic sensor measures the temperature increase caused by incident ultrasound energy to determine the ultrasound intensity. However, the sensor characteristics are not only dependent on applied ultrasound intensity, but also on ambient temperature and the slightly changing acoustic properties of absorber materials as the absorber heats up, which create a complex problem in sensor design. To obtain an accurate and consistent measurement, these effects should be considered and compensated for in the thermal sensor design. The traditional computational method usually identifies a deterministic mathematical relationship through data interpolation; however, this method is inadequate for solving the problem, since it is extremely difficult to resolve the mathematical formula, if not impossible, among multiple confounding variables such as the temperature change of the sensor, applied ultrasound intensity and ambient temperature from measured data. We previously showed a solution using extrapolation and interpolation based on calibration values [19], but the method is still unsatisfactory due to the requirement of complex calibration, calculation procedures, and limited improvement in accuracy. In this paper, we propose the implementation of an artificial neural network to identify the relationship and solve the problem. An artificial neural network can map the implicit relationship of inputs and outputs through the training and testing of measured data, which has been applied to compensate for the various nonlinear errors in system designs [20,21,22,23,24]. Through proper training, the artificial neural network can compensate for the nonlinear errors, enabling a direct read-out of the applied ultrasound intensity.

## 2. Sensor Design and Simulation

### 2.1. Sensor Design

**Figure 1:**
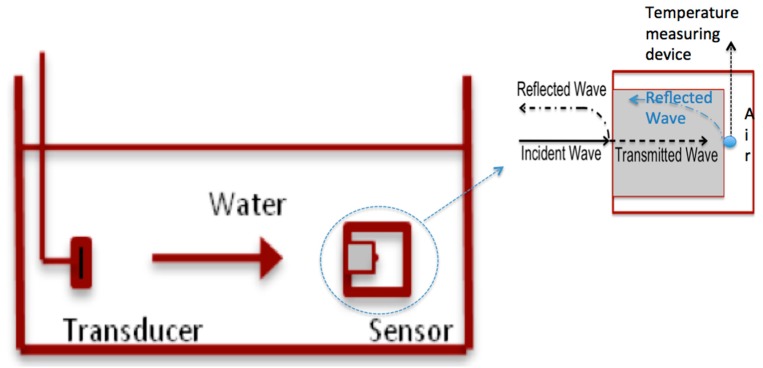
Schematic of the standard thermoacoustic sensor and its setup.

To design a sensor for measuring I_sata_, adoption of the conventional beam-plotting set-up would require a complicated set-up procedure (shown in Figure 1) involving the suspension and alignment of the sensor and transducer in a water tank, with a solid cylindrical absorber in the sensor to convert the incident ultrasound energy into heat. A mechanical positioning system is required for the accurate positioning and alignment of the transducer and the sensor in the water tank.

The ultrasound beam propagates through water and reaches the front end of the sensor. In single reflection approximation, by ignoring a small portion of the reflected ultrasound wave between the medium and sensor (refer to Figure 1), almost all transmitted ultrasound energy is being absorbed and converted into heat by the material when the waves transmit through the absorber (The ultrasound wave is fully reflected by the back wall of the absorber due to large impedance mismatch of the absorber-air interface). Any reflected ultrasound waves return to the water through the front face of the sensor [15]. The sensor design estimates the ultrasound intensity by the amount of temperature increase caused by absorbed heat.

Either a commercial positioning system or a customized positioning system would increase the cost of our senor design. Without such a mechanical positioning system, it is quite difficult to totally align the absorber inside the sensor with the transducer in a water tank during each measurement. Any misalignment of the sensor’s absorber with the transducer during a measurement compromises the accuracy of the sensor’s measurement and makes it difficult to guarantee measurement consistency. Therefore, by directly coupling the sensor with a transducer through an ultrasound medium layer, our close-proximity sensor design not only simplifies the set-up, but also guarantees the consistency of each measurement without a positioning system. Our previous work showed that our design took 20 s to conduct measurements, and that the maximum measurement error was less than 11% in 18 measurements, which is a much more convenient and efficient measurement technique by comparison to the conventional set-up [19]. However, to achieve much greater measurement efficiency, further design modifications are required.

**Figure 2:**
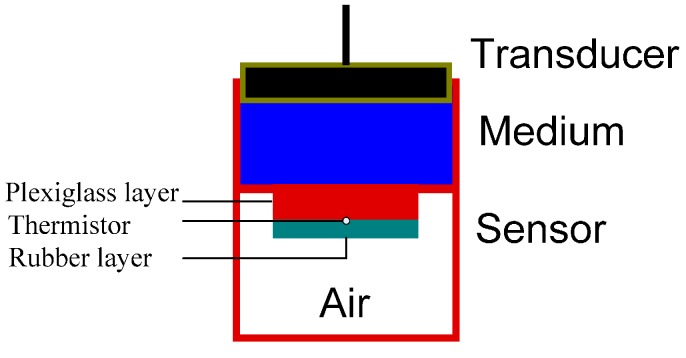
The structure of the two-layer thermoacoustic sensor.

The energy absorption rate of the sensor is important for accurate and rapid estimation of ultrasound intensities because higher absorption rates indicate: (1) more reliable estimations based on the amount of captured energy; and (2) more rapid measurement readout due to faster energy conversion rates. We propose to use a two-layer structural design as well as better absorbing materials to increase the efficiency of our sensor. Figure 2 illustrates the structure of the new two-layer sensor.

In our previous design, a single layer of plexiglass was used for the absorber because it is a low-cost material with easy-to-process mechanical properties and a relatively high attenuation coefficient of 1.13 dB·cm^−1^ MHz^−1^. For the new design, we added another absorber layer to the back of the plexiglass layer, made of a highly attenuating polyurethane rubber APTFLEX F28 (Precision Acoustics Inc. Dorchester, UK), in order to further increase the absorption of ultrasound energy. The thicknesses of the plexiglass and rubber layers are both 1 mm, and the diameter of the absorber is 20 mm. The gap between the two absorber layers is filled by a very thin film of acoustically matched material APTFLEX F21 (Precision Acoustics Inc.). This film ensures no air is trapped between the layers, otherwise it causes an air-absorber impedance mismatch that reflects ultrasound waves before entering the second layer. Two-layer design facilitates a smooth ultrasound transition from one layer to the next. This new sensor design adopts the close-proximity concept to simplify set-up procedures through the use of direct coupling between sensor and transducer via coupling medium (for details about the close-proximity design, please refer to [19]). A micro thermistor (0.36 mm in diameter, Honeywell Inc. Morristown, NJ, USA) is used to measure temperature changes of the sensor. Silver particles are applied in the two-layer interface to equally distribute the generated heat around the thermistor. A 0.3 mm thick layer of copper is employed in order to distribute heat in our previous one-layer design [19]. However, in our new design, its high reflection coefficient prevents ultrasound propagation from one layer to the next and is therefore replaced by silver particles. In terms of thermal diffusivity (representing the ability of a material to redistribute heat), silver has a value of 1.74 × 10^−4^ m^2^/s which is greater than that of copper at a value of 1.15 × 10^−4^ m^2^/s [25]. The temperature increase measured by the thermistor is sent to a microcontroller for real-time processing and recording. The absorber is sealed in the sensor in order to reduce any outside interference. After sealing the absorber, the ultrasound medium temperature becomes the starting ambient temperature that affects the sensor’s measurement accuracy, since the ultrasound medium is in contact with the absorber.

### 2.2. Simulation of Ultrasound Propagation in Sensor

To evaluate the performance of the two-layer sensor, 3-D simulation models of the ultrasound propagation in the sensors were developed using a K-wave toolbox (Version 1.1). K-wave is an open source Matlab and C++ toolbox designed for time domain ultrasound simulation [30,31]. The 3-D models for the one-layer and two-layer sensors were separately built based on sensor structure, acoustic properties of media, and materials used in the designs. Table 1 lists the acoustic properties of the media and materials included in the 3-D models. For the one-layer sensor model, the ultrasound wave propagates through the ultrasound medium and plexiglass absorber, and then reflects back at the absorber-air interface whereas, in the two-layer sensor model, the ultrasound wave propagates from the first plexiglass layer to a second polyurethane layer after transmitting from the ultrasound medium into the sensor. The simulation of models in K-wave is implemented by dividing the built model into tiny computational grid points and computing the propagation of the wave field point by point. To accurately simulate ultrasound wave propagation, a high-resolution grid spacing of 0.05mm is chosen in ultrasound propagation direction. A simulated planar detector array is set to measure and record output ultrasound pressure during the simulation. The simulation compares the amount of absorbed ultrasonic energy between the two-layer sensor and that of the one-layer sensor.

**Table 1:** Acoustic properties of media and materials (20 °C) [26,27,28,29].

Material	Attenuation Coefficient (dB·cm^−1^ MHz^−1^)	Density (kg/m^3^)	Speed of Sound in Media (m/s)
Ultrasound Medium	0.002	1000	1481
Air	1.64	1.204	343
Plexiglass	1.13	1180	2730
Polyurethane Rubber	30	1010	1500

**Figure 3:**
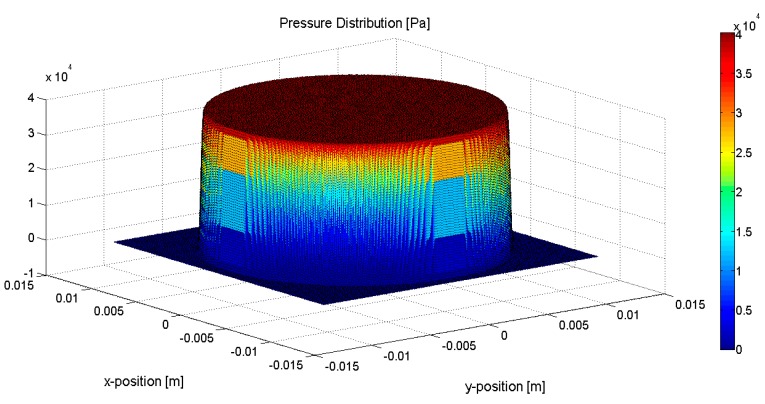
Pressure distribution of the generated ultrasound wave.

The same ultrasound wave in our 3D model is generated and transmitted to sensors. The pressure distribution of the ultrasound wave is shown in Figure 3, where the X and Y axes represent spatial coordinates on the sensor’s surface, and the Z axis represents the ultrasound pressure which is scaled using jet colormaps. The ultrasound wave is a plane-wave, which in turn is an approximation of the realistic ultrasound wave generated by the transducer of the Sonacell ultrasound generator.

The majority of the ultrasound energy is transmitted into the sensors, reflected at the back of the sensor, and then propagates through the front face back to the medium. In the simulation, the remaining ultrasound energy after passing through each sensor is measured by the planar detector array. Acoustic attenuation of a material is represented by Equation (1):

(1) $$I\left(x\right)=I_{0}e^{-\text{α}fx}\;$$

where *I_0_* represents the incident ultrasound intensity (mW/cm^2^), *I(x)* is the ultrasound intensity after attenuation, *x* is the thickness of the material, α is the attenuation coefficient of the material and *f* is the frequency of the incident ultrasound.

**Figure 4:**
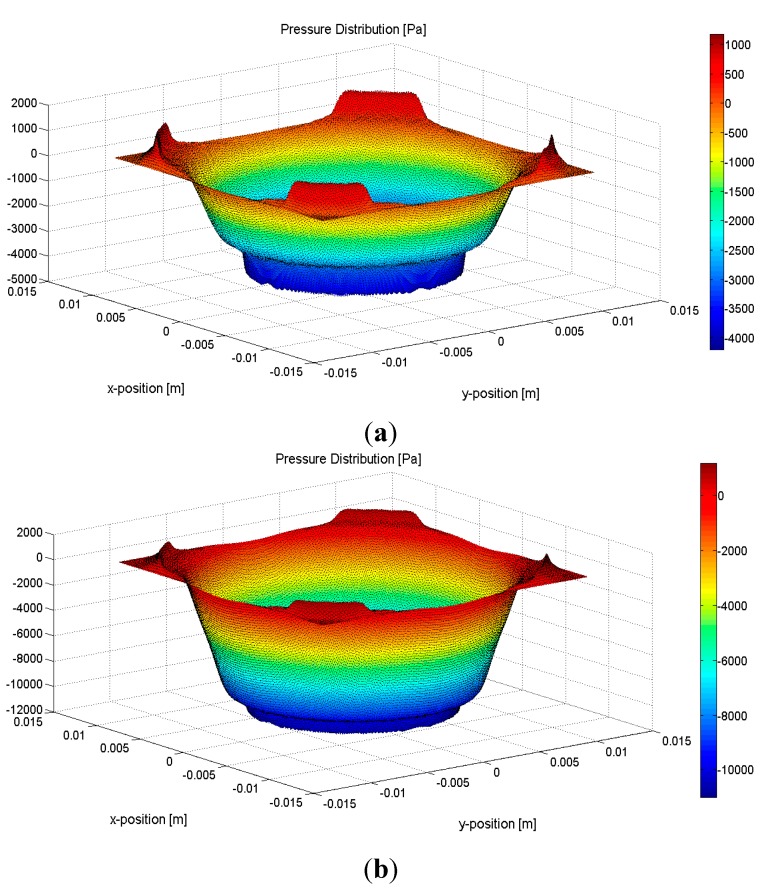
Ultrasound pressure distributions of the remaining waves for (**a**) the two-layer sensor after attenuation, and (**b**) the one-layer sensor after attenuation.

Equation (1) shows that acoustic attenuation is not only a function of a material property, but also a function of frequency. The absorption rate of the sensor therefore increases with the increasing acoustic frequency due to the frequency-dependence of the absorber materials acoustic attenuation. The simulation is based on an acoustic frequency of 1.5 MHz, which has been widely used in therapeutic applications. Figure 4a,b shows the ultrasound pressure distributions of the remaining waves after attenuation by the two-layer and one-layer sensors respectively, where the *X* and *Y* axes represent section position, and the *Z* axis represents the ultrasound pressure of the corresponding position.

The absorption efficiency of the two sensors is evaluated by calculating and comparing the average pressure of the remaining waves after attenuation by the sensors with the average pressure of the input wave. The evaluation results show that approximately 7.5% and 22.5% of the input ultrasound energy are transmitted after passing through the two- and one-layer sensors respectively, thus establishing the two-layer sensor as the better of the two.

## 3. Sensor Calibration

### 3.1. Calibration for Thermistor Data

After making the sensor, a quantitative relationship between the temperature of the sensor and the readout of the thermistor becomes imperative; thus, a quadratic model is adopted:

(2) $$\text{y}\left(x\right)=ax^{2}+bx+c$$

where *y(x)* is the sensor temperature (°C) and *x* is the digital readout of the thermistor.

Thermistor calibration is carried out by placing the sensor in a heated water bath in order to determine the value changes of the thermistor with respect to changes in temperature. The rising sensor temperature is recorded by a thermocouple with a sensitivity of 0.1 °C, while the corresponding voltage value of the thermistor is measured and converted into a digital number through a microcontroller’s analog to digital converter.

**Figure 5:**
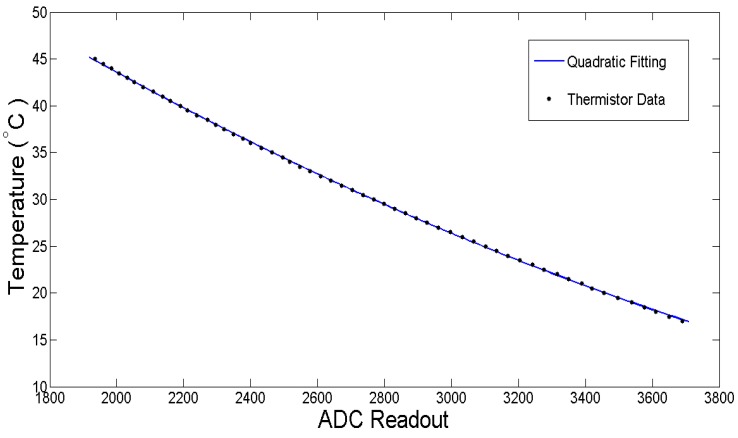
Thermistor curve fitting based on the quadratic model.

MATLAB’s curve fitting toolbox is used to find best estimates for the constants *a*, *b*, and *c* in Equation (2). The final parameter values obtained from curve fitting are *a* = 2.25 × 10^−6^ °C, *b* = −0.284 °C, *c* = 91.42 °C. Figure 5 shows the curve fitting results of the measurement data based on the quadratic model. Table 2 shows the curve fitting accuracy parameters.

**Table 2:** Curve fitting accuracy of thermistor calibration.

	SSE	R-squared	RMSE
Quadratic Model	0.3335	0.9999	0.07859

SSE is the sum of squared errors between the best-fit curve: the smaller the SSE value, the better the model fits the data. R-squared represents the percentage of the sum of squares in a data set accounted for by the model. A value of R-squared equal to 1 indicates the model fits the data perfectly. The root-mean-square error (RMSE) provides a measure of the difference between values predicted by the model and the observed values. From Figure 5 and Table 2, it is concluded that the quadratic model exhibits excellent performance for thermistor data fitting.

### 3.2. Approach for Relating Temperature Rise to Ultrasound Intensity

The equilibrium temperature approach and transient temperature profile are two methods for relating a temperature increase to an applied ultrasound intensity. Most of the previous thermoacoustic designs employed an equilibrium temperature approach that balances the heat generated by ultrasound energy and the heat expelled to the surrounding medium [13,14,15]. However, it takes a long time to reach thermal equilibrium which in turn prevents a rapid determination of ultrasound intensities. To provide a rapid measurement, we employed a transient temperature method, which quickly estimates the applied ultrasound intensity given the transient temperature increase in the sensor. Such an approach enables rapid readouts of the applied ultrasound intensity based on a temperature-rise coefficient [17,18,19]. The first mode of transient profiles is given by Equation (3).

(3) $$\text{T}\left(\text{t}\right)={\text{T}}_{\text{s}}+\text{C}\left(1-{\text{e}}^{\frac{-\text{t}}{\text{τ}}}\right)$$

where *T(t)* is the measured temperature of the absorbing cylinder, *T_s_* is the starting temperature, *C* represents the temperature-rise coefficient determined by the ultrasound intensity, *τ* is a time constant determined during curve fitting procedure and *t* represents measurement time.

**Figure 6:**
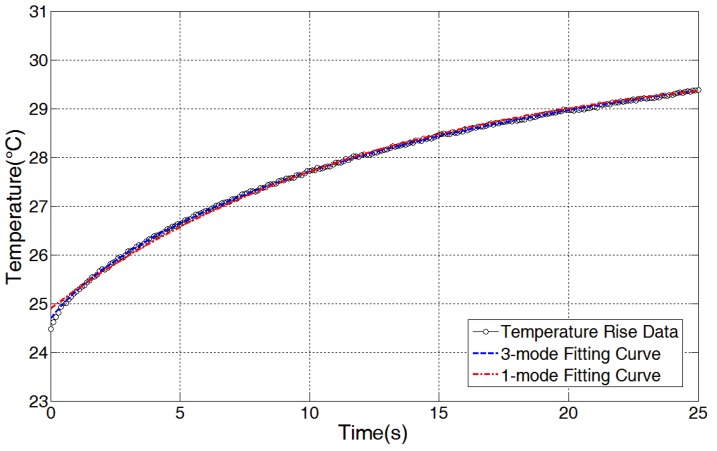
Curve fitting results for temperature rise data by MATLAB’s curve fitting box (60 mW/cm^2^).

Data fitting is performed using MATLAB’s curve fitting toolbox. Figure 6 shows the curve fitting results for temperature increases in the sensor with an ultrasound intensity of 60 mW/cm^2^. An ultrasound intensity of 60 mW/cm^2^ is generated by the Sonacell generator and calibrated using a radiation force balance (UPM-DT-1AV, Ohmic Instruments. St. Charles, MO, USA). Table 3 shows the coefficients of the fitting curve based on Equation (3), and Table 4 shows its curve fitting accuracy for the temperature rise data. From Figure 6 and Table 4, we conclude that the fitting curve based on Equation (3) does conform to the measured data, which validates the feasibility of the transient temperature model in this sensor design. It is worth noting that an obvious deviation of the first-mode fitting curve from the measured data exists at the very beginning in Figure 6. Reference [18] shows that three modes of transient profiles can improve the fitting accuracy of the steep rising part at the very beginning, and therefore, the fitting curve based on the three modes is also included in Figure 6 for a comparison. The three modes of transient profiles are represented by Equation (4):

(4) $$\text{T}\left(\text{t}\right)={\text{T}}_{\text{s}}+{\text{C}}_{1}\left[\left(1-e^{\frac{-t}{\tau_{1}}}\right)-{\text{C}}_{2}\left(1-e^{\frac{-t}{\tau_{2}}}\right)+{\text{C}}_{3}\left(1-e^{\frac{-t}{\tau_{3}}}\right)\right]$$

where *τ_1_*, *τ_2_ and τ_3_* are the time constants, *C_2_* and *C_3_* are the coefficients and *C_1_* is the only coefficient related to the ultrasound intensity.

Figure 6 shows that the fitting curve based on the three modes of transient profiles does have a better approximation of measured data of the steep rising part at the very beginning. In our sensor design, the approach based on Equation (4) was not used, since the approach makes data evaluation and calibration more complex, and especially since the embedded system based microcontroller has great difficulty accurately fitting the temperature data based on the model in real-time due to its complexity. Curve fitting based on Equation (3) suggests that the coefficient *C* at a specific ambient temperature is directly related to the applied ultrasound intensity; this relationship is applied to the following neural network training.

**Table 3:** The coefficients of the first transient model curve fitting.

Ultrasound Intensity (mW/cm^2^)	Coefficient C (°C)	Coefficient τ (s)	Coefficient T_0_ (°C)
60	4.847 (4.798,4.895)	10.75 (10.46,11.04)	24.9 (24.88, 24.93)

Note that the numbers in the brackets show prediction bounds with 95% certainty.

**Table 4:** Curve fitting accuracy of the first transient model curve fitting.

SSE	R-square	RMSE
0.4923	0.9982	0.04999

## 4. Artificial Neural Network in Sensor Design

### 4.1. Artificial Neural Network Model in Sensor Design

**Figure 7:**
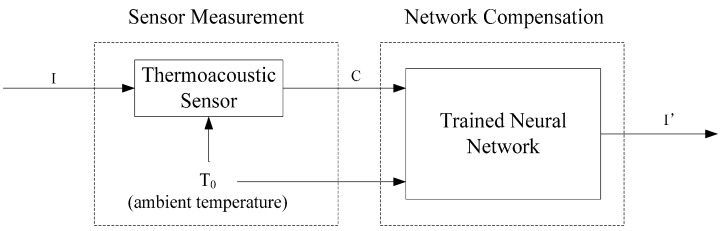
Model of the thermoacoustic sensor compensation using a neural network. The neural network algorithm is implemented using a microcontroller.

For each of the various discrete values of the starting ambient temperature and intensity, the thermal characteristic of the sensor is stable and its coefficients through curve fitting are unique. The identification of the relationship among the parameters is the basis of the thermoacoustic sensor design. It is extremely difficult to use traditional computational methods to resolve the mathematical formula and identify the relationship. To solve this problem, an artificial neural network method is introduced to map the relationship and compensate for temperature drifts. The compensation model using neural networks is shown in Figure 7.

In this model, *I* is the applied ultrasound intensity to be measured. *T_0_* is the ambient temperature that affects the sensor measurement accuracy. *C* is the temperature rising coefficient calculated by the sensor, which is affected by the ambient temperature. *I’* is the adjusted ultrasound intensity after neural network compensation. The proper parameters of the neural network are determined by training with 50 pairs of measurements (refer to our later discussion in Section 4.2). The trained neural network allows for both rapid and accurate estimations of *I* based on *C* and *T_0_*.

### 4.2. Artificial Neural Network Training

**Figure 8:**
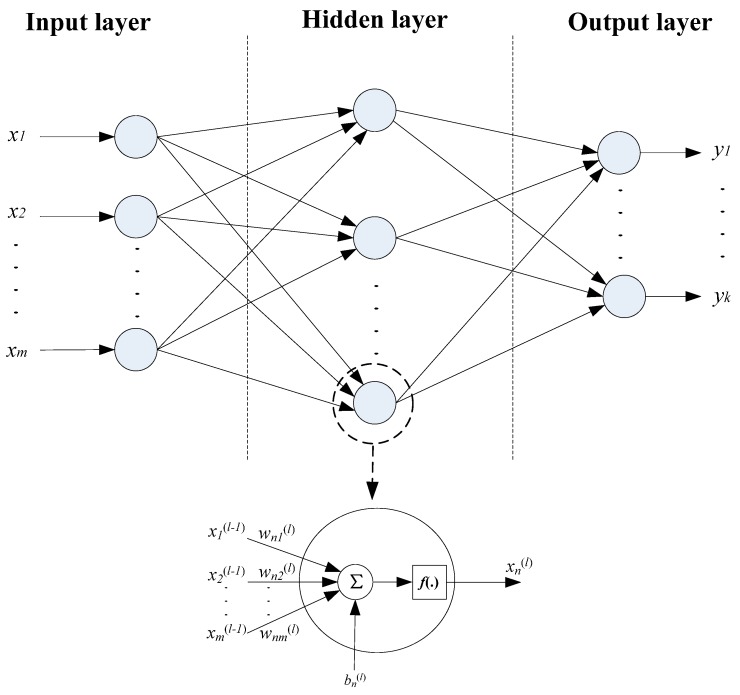
Schematic of a three-layer artificial neural network.

The back propagation (BP) neural network is one of the most widely used artificial neural network models based on a multi-layer perceptron structure and a gradient descent optimization algorithm [20]. The algorithm calculates gradient descent and iteratively updates network weights in order to minimize error between the network output and the desired output. The artificial neural network consists of three layers: the input layer, one or more hidden layer(s), and the output layer. An illustrative three-layer artificial neural network is shown in Figure 8. As illustrated in the figure, each layer has a number of nodes (neurons) that fully connect to adjacent layers, and each node receives the outputs from the previous layer with a distinct set of weights.

A three-layer neural network (Figure 8) is applied in our sensor design and trained through the back propagation algorithm. The number of nodes in the input layer, hidden layer, and output layer were chosen to be 2, 3, and 1, respectively. To target low ultrasound intensities commonly set for biomedical applications at regular room temperatures, we take measurements on a range of intensities within 30 mW/cm^2^ to 120 mW/cm^2^ with an increment of 10 mW/cm^2^ at ambient temperatures 20 °C, 21.5 °C, 23 °C, 24.5 °C and 26 °C. The values of *C* corresponding to each ultrasound intensity and room temperature were obtained through curve fitting. The three parameters of *C*, ultrasound intensity, and ambient temperature comprise a data set, and a total of 50 data sets were obtained and recorded for network training.

**Figure 9:**
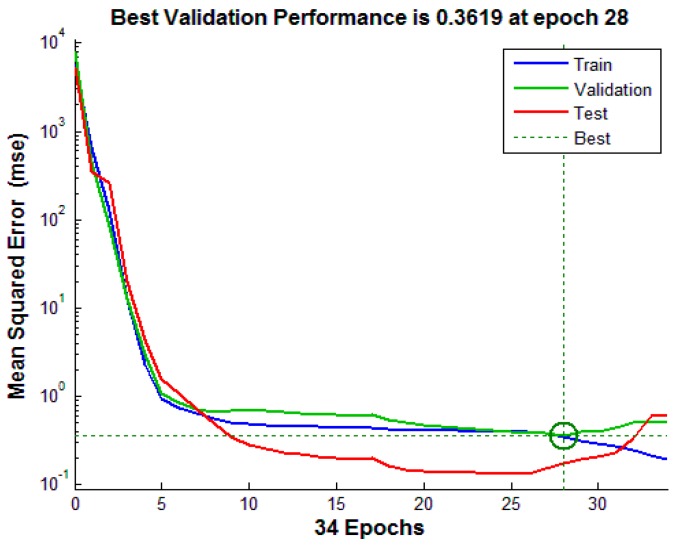
Variation of mean squared error with training epochs.

The neural network training is implemented through the MATLAB Neural Network toolbox. 88% of the total data sets were randomly selected for training, 6% for validation and 6% for testing. The training data is used to update network weights and biased interactively based on the gradient calculations; application of all training data sets to train the network constitutes one training epoch or iteration. The training performance after each epoch is evaluated using mean squared error between the current estimation and the target output. The generalization error of the validation sets is monitored during the training process and used to decide when to stop training. Although test data sets are not involved in the training process, the error of test data sets is useful to independently evaluate the training performance. The training process continues epoch after epoch until a best validated performance is found. Figure 9 shows variations of mean squared error with training epochs. The errors of the training data, validation data, and training data keep decreasing at the beginning stage of training. Although the error of the training group still decreases after epoch 28, the errors of the validation and testing groups begin to increase, which indicates the network has been over-trained after epoch 28. Therefore, the trained network has the best validated performance at epoch 28. The weight and bias matrices for the trained neural network at epoch 28 are shown in Equations (5)–(8), which were saved and programmed into the micro-controller in order to realize the temperature compensation. *W_ih_* denotes the weight between the input layer and the hidden layer, *W_ho_* denotes the weight between the hidden layer and the output layer, *B_h_* and *B_o_* represent the bias of the hidden layer and the output layer respectively.

(5) $$W_{ih}=\left[\begin{array}{ccc} 3.5008 & 5.0117 & 0.47412\\ -1.3035 & 4.4669 & -0.10647 \end{array}\right]\;$$

(6) $$\;B_{h}\;=\left[\begin{array}{ccc} -3.3727 & 1.8568 & 0.086394 \end{array}\right]\;$$

(7) $$\;W_{ho}={\left[\begin{array}{ccc} 0.1084 & 0.014241 & 2.8166 \end{array}\right]}^{T}$$

(8) $$\;B_{o}\;=\left[-0.079312\right]$$

**Figure 10:**
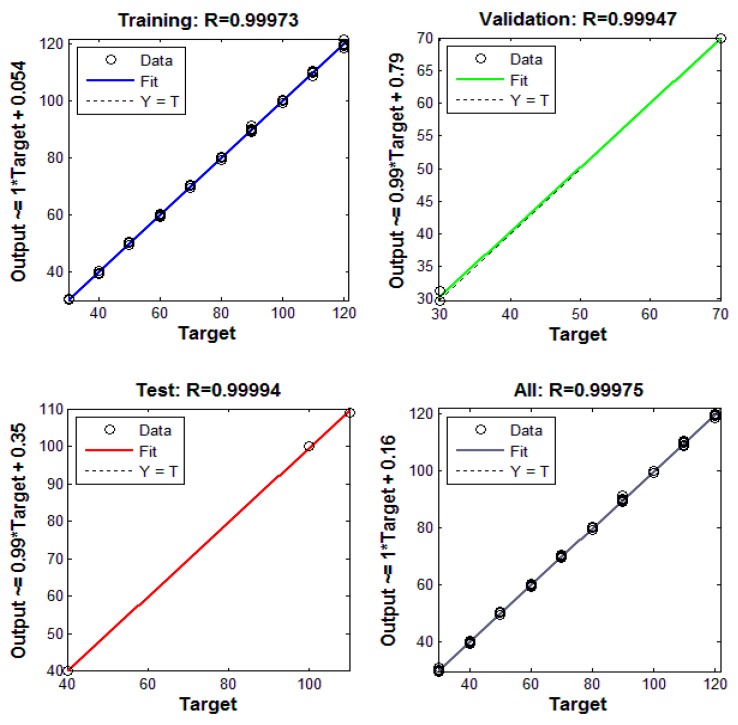
The agreement between the network’s output intensity and target intensity.

The network training results for training data, validation data, testing data, and all data are shown in Figure 10.

The performance of the trained network is evaluated by comparing its output intensity based on the input of *C* and ambient temperature with the real applied intensity (target intensity). Regardless of various ambient temperatures and applied intensities, a very good agreement between the network’s output intensity and target intensity for all data can be observed from the figure; the estimated output intensity matches the target intensity very well. The results verify that the trained neural network can effectively correct ambient temperature effects and accurately measure the ultrasound intensity.

## 5. Sensor Performance Evaluation

### 5.1. Neural Network Evaluation with Untrained Data Sets

**Figure 11:**
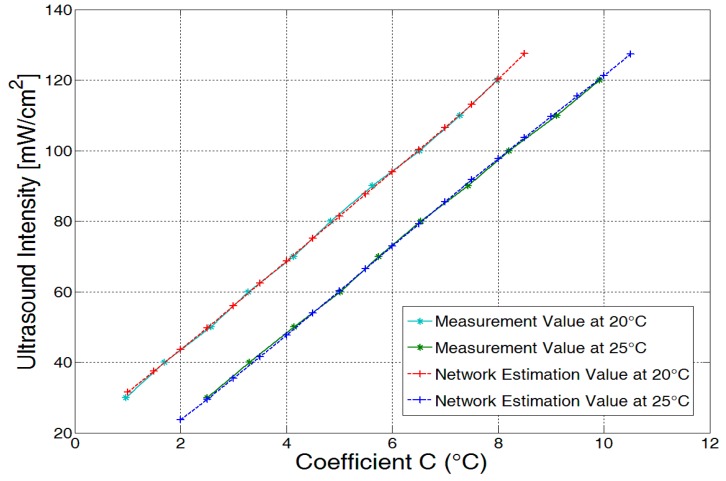
Comparison between the estimated data sets and the real measurement data sets.

The performance of the trained network is further evaluated at two ambient temperatures on *C* that has never been trained and tested. At 20 °C, a set of *C* from 1 to 8.5 in 0.5 increments are given to the neural network, and estimated ultrasound intensities are generated as output. At 25 °C, a temperature that has never been trained, another set of *C* from 2 to 10.5 in 0.5 increments were fed into the neural network. The estimated ultrasound intensities and real measurements were compared with each other in order to evaluate the performance of the neural network. The estimated and real measured data sets at temperatures of 20 °C and 25 °C were plotted in Figure 11. As illustrated in the figure, the estimated intensities and the real measurements almost entirely overlap at both temperatures, thus verifying that the trained network not only works for trained data sets, but is also valid for untrained data sets.

### 5.2. Network Temperature Compensation Performance

**Figure 12:**
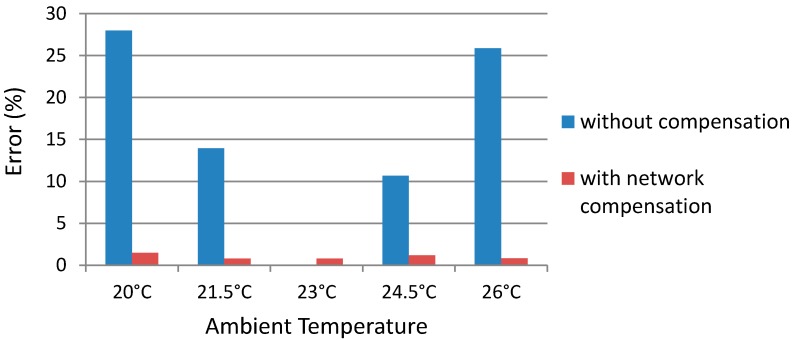
Ultrasound intensity error with and without network temperature compensation.

To evaluate the performance of network temperature compensation, a comparison of the measurement results with and without the network temperature compensation is conducted. Figure 12 shows measurement errors with and without network temperature compensation over a range of ambient temperatures at an intensity of 50 mW/cm^2^. The measurement error with network compensation is the difference between the applied intensity and the trained network output intensity, while the measurement error without compensation is obtained by comparing the applied intensity with the estimated intensity based on values of the corresponding *C* and the reference *C* at a temperature of 23 °C. The evaluation results show that the average error with network compensation is around 1%, while the average error without network compensation is above 15%. The measurement error with network compensation is insensitive to the temperature drift, while the measurement error without network compensation increases with the temperature drift.

### 5.3. Sensor Response Time

**Figure 13:**
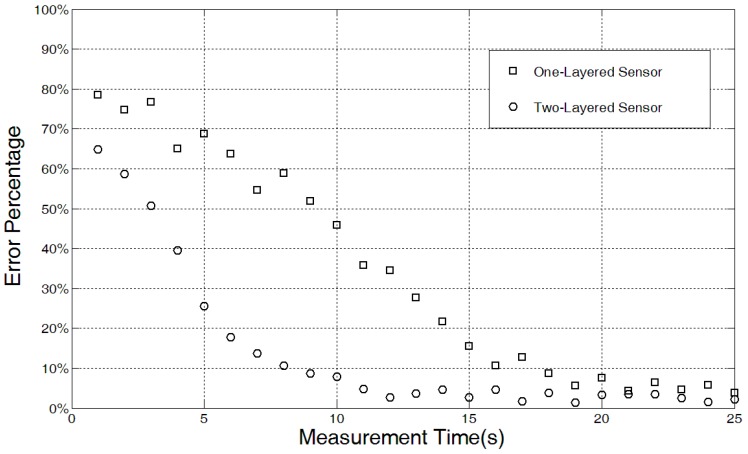
Response time of the one- and two-layer sensors with respect to measurement error percentage.

It is important to design a sensor capable of producing an accurate measurement in a short response time. The new two-layer structure improves the absorption efficiency of the sensor design, and provides a more rapid and reliable estimation of ultrasound intensity based on faster energy conversion rates and a greater amount of captured energy. To evaluate the improvement of the two-layer sensor design, the response time of the one- and two-layer sensors were measured and evaluated. Figure 13 shows the measurement error percentage of the sensor designs at different time points under an ultrasound intensity of 40 mW/cm^2^.

The error is the difference between the target intensity and the value measured by thermoacoustic sensors based on curve fitting. By inspecting the figure, we can determine the response time required for the two types of sensors to obtain a reliable measurement. The two-layer sensor can provide a reliable measurement in approximately 12 s, whereas it takes roughly 20 s for the one-layer sensor to obtain an acceptable measurement.

### 5.4. Measurement Comparison with Our Previous Design

**Figure 14:**
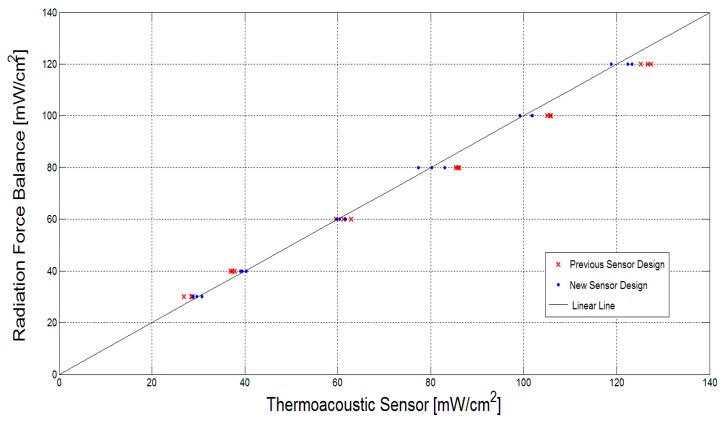
Comparison of the new sensors design measurements with that of the radiation force balance as a means to conduct a performance evaluation.

To extensively evaluate the improvement in measurement accuracy of the new two-layer sensor design, the SonaCell ultrasound generator is calibrated by the radiation force balance (UPM-DT-1AV, Ohmic Instruments, St. Charles, MO, USA) in an environment without noise vibrations in order to generate ultrasound intensities of 30, 40, 60, 80, 100 and 120 mW/cm^2^. The intensities are derived by dividing the measured power by the beam area of 3.5 cm^2^, which was directly measured by a needle hydrophone system (Precision Acoustics Inc. Dorchester, UK) [32]. The intensities generated by the SonaCell generator are constant at room temperature. The new designed sensor was employed to measure each intensity at ambient temperatures of 22 °C, 23 °C, and 24 °C in order to investigate the agreement of the two techniques. Table 5 shows measurement results of ultrasound intensities obtained by the new sensor design based on the artificial neural network. The measurement results given by the previous one-layer sensor design in [19] are also included for better comparison. In the previous sensor design, a temperature compensation method through extrapolation or interpolation based on the calibration values was used to estimate the applied ultrasound intensities. All the measurement results are plotted in Figure 14 for comparison and evaluation. In the figure, the linear fit represents a 1:1 relationship between the radiation force balance and the thermoacoustic sensor. The excellent measurement agreement between the new sensor design and the radiation force balance technique confirms that the sensor’s measurements are accurate. Over the 18 measurement samples, the previous design has an average error of 3.97 mW/cm^2^, while the new sensor design has an average measurement error of 1.31 mW/cm^2^. Root-mean-square error (RMSE) of the previous design is 4.49, whereas RMSE of the new sensor design is 1.63. Therefore, by evaluating the average error and RMSE of the measurement results, the great improvement in measurement accuracy of the new sensor design has been demonstrated.

**Table 5:** Measurement results of the new sensor design.

Target *I* (mW/cm^2^)	Thermoacoustic Sensor *I* (mW/cm^2^)		
	#1	#2	#3
30	28.85	30.74	29.68
40	40.33	39.42	39.06
60	61.66	60.37	59.82
80	77.33	80.22	83.03
100	101.78	99.12	101.9
120	123.26	118.86	122.43

## 6. Discussion

A novel two-layer thermoacoustic sensor based on an artificial neural network as a means to adapt to temperature drifts was proposed, implemented, and investigated in order to measure low ultrasound intensities. Compared to our previous sensor design where only one layer of plexiglass absorber is used, the new sensor design employed two absorber layers with a plexiglass layer in the front and a rubber layer at the back. The current design has improved the sensor’s absorption efficiency, as demonstrated by simulation, and thus resulting in a more rapid and reliable estimation of ultrasound intensity. The two-layer sensor design demonstrates that sensor performance can be improved by optimizing its physical structure.

The temperature increase of the sensor depends not only on the ultrasound intensity, but also ambient temperature and the slightly changing acoustic properties of absorber materials that change with temperature, which in turn makes sensor measurements a complicated inverse problem. It is extremely difficult to resolve the exact mathematical relation. To overcome this difficulty, the method of artificial neural networks is proposed and applied into the thermoacoustic sensor design. Application of the artificial neural network method requires training the data in a proper manner. The data are divided into two groups. Most of the data are used to directly train the network, while the other data are set aside for validation and testing purposes. If all the data are used to train the network, the problem of over-training appears, in which the trained network works well for the training data with minimum error, but cannot be generalized to the untrained data well. Therefore, some data are set aside to check for the presence of over-training and to decide when to stop training in order to obtain an optimal network that can minimize the generalization error. Through proper training, the artificial neural network can compensate for measurement errors caused by temperature drifts and enable the sensor to directly measure the applied ultrasound intensity. The experimental results demonstrate that the trained network not only validates on the data sets with training, but also is adaptive to the untrained data sets in the training range. The experimental results show that measurement error is reduced from greater than 15% without network compensation, to 1% with network compensation. The current design trained the network to adapt to room temperatures ranging from 20 °C to 26 °C, however the adaptive temperature range of the sensor design can easily be extended by training more data sets in a wider temperature range.

Thermocaoustic sensors, due to their relatively simple structure, have the advantage of low cost and simplicity over the other techniques used to measure ultrasound intensities. Our design is based on the close-proximity sensor concept as a means to measure spatial-average temporal-average intensity (I_sata_), which not only simplifies the set-up, but also guarantees the consistency of each measurement. For the I_sata_ measurement, radiation force balance is a benchmark technique and has a minimal measurement uncertainty (3%) without vibration noises. The radiation force balance is used to calibrate the ultrasound generator for the sensor design, which would link the thermoacoustic sensor’s measurement error to the radiation force balance’s uncertainty. In addition, the uncertainty of the beam area used to derive ultrasound intensity also contributes to the absolute calibration uncertainty of the thermoacoustic sensor, and therefore, our sensor design cannot provide more accurate measurements than the radiation force balance in a vibration-free environment. However, in an environment with vibration noises, such as in a biology laboratory, the error of a radiation force balance can easily surpass 20% for low ultrasound intensity measurements, whereas our sensor design only has an overall measurement uncertainty of 5%. For thermoacoustic sensors, it is better to let the sensor cool down before beginning the next measurement in order to maintain measurement accuracy. However, the cool-down time between measurements should not be considered as a disadvantage for the thermoacoustic sensor design, since the radiation force balance, as the benchmark technique, also needs a short period of time between measurements while it waits for a force balance. It takes around 10~15 s for the radiation force balance to take a measurement and 10~20 s between measurements to wait for a force balance to settle down, while it takes 12 s for our sensor to take a measurement and around 13 s for the sensor to cool down between measurements. Table 6 shows advantages and disadvantages of both techniques for further comparison.

**Table 6:** Advantages and disadvantages of radiation force balance and the thermocaoustic sensor.

	Advantages	Disadvantages
Radiation Force Balance	A benchmark technique with minimal measurement uncertainty in a vibration-free environment Versatile for a range of transducers in a wide intensity range	Cannot provide accurate measurements with noise vibrations Added complexity when measuring individual transducers in a transducer array High cost
Thermoacoustic Sensor	Measurements not affected by noise vibration An easy-to-operate device for measuring individual transducers in a transducer array Low cost	A calibration process is needed for a particular transducer Designed for low intensity ultrasound

The designed thermoacoustic sensor can provide a real-time measurement and process the sensor’s temperature change, since a microcontroller is used in the sensor’s design to form an embedded system. By measuring and processing temperature data, the value of ultrasound intensity is obtained in 12 s. The microcontroller is implemented in a printed circuit board outside the sensor to process the temperature data sent by the thermistor and to perform compensations based on the artificial neural network. In the future design, the microcontroller can be integrated into the ultrasound generator system in order to measure and control the output intensity simultaneously, which makes the ultrasound auto-calibration system become a possibility.

## 7. Conclusions

A novel two-layer thermoacoustic sensor based on an artificial neural network is described and investigated in this paper. The structure design improvement enables the sensor to reduce its measurement time from 20 s to 12 s. The artificial neural network algorithm is integrated in order to compensate for the influence of the temperature drifts, adapting the sensor for a range of ambient temperatures, and providing an accurate and consistent measurement of the ultrasound intensities. The experimental result show that the compensation provided by the artificial neural network reduced the temperature drift errors from more than 15% to 1%. The final results show that the new sensor achieves an average error of 1.31 mW/cm^2^ over 18 measurement samples.

The new sensor design is a low-cost alternative method that can provide rapid ultrasound intensity measurements without any complex set-up procedure. Although the radiation force balance is the golden standard method with minimal measurement error, the application of the technique is limited by the requirements of experience in regards to equipment set-up and operation. In addition, measurement accuracy is affected by background vibrations, which in turn limits its application in biology laboratories. The new thermoacoustic sensor design is an easy-to-operate alternative method for rapidly measuring low ultrasound intensity (30 mW/cm^2^ to 120 mW/cm^2^) with high accuracy, especially in a practical environment like a biology laboratory.
